# Replication of Associations of Genetic Loci Outside the HLA Region With Susceptibility to Anti–Cyclic Citrullinated Peptide–Negative Rheumatoid Arthritis

**DOI:** 10.1002/art.39619

**Published:** 2016-06-24

**Authors:** Sebastien Viatte, Jonathan Massey, John Bowes, Kate Duffus, arcOGEN Consortium, Stephen Eyre, Anne Barton, Jane Worthington, John Loughlin, Nigel Arden, Fraser Birrell, Andrew Carr, Panos Deloukas, Michael Doherty, Andrew W. McCaskie, William E. R. Ollier, Ashok Rai, Stuart H. Ralston, Tim D. Spector, Ana M. Valdes, Gillian A. Wallis, J. Mark Wilkinson, Eleftheria Zeggini

**Affiliations:** ^1^ Arthritis Research UK Centre for Genetics and Genomics, Manchester Academic Health Science Centre, and University of ManchesterManchesterUK; ^2^ NIHR Manchester Musculoskeletal Biomedical Research Unit, Central Manchester NHS Foundation Trust, Arthritis Research UK Centre for Genetics and Genomics, Manchester Academic Health Science Centre, and University of ManchesterManchesterUK

## Abstract

**Objective:**

Genetic polymorphisms within the HLA region explain only a modest proportion of anti–cyclic citrullinated peptide (anti‐CCP)–negative rheumatoid arthritis (RA) heritability. However, few non‐HLA markers have been identified so far. This study was undertaken to replicate the associations of anti‐CCP–negative RA with non‐HLA genetic polymorphisms demonstrated in a previous study.

**Methods:**

**The Rheumatoid Arthritis Consortium International densely genotyped 186 autoimmune‐related regions in** **3,339 anti‐CCP–negative RA patients and 15,870 controls across 6 different populations using the Illumina ImmunoChip array. We performed a case–control replication study of the anti‐CCP–negative markers with the strongest associations in that discovery study, in an independent cohort of anti‐CCP–negative UK RA patients. Individuals from the arcOGEN** **Consortium and Wellcome Trust Case Control Consortium were used as controls. Genotyping in cases was performed using Sequenom MassArray technology. Genome‐wide data from controls were imputed using the 1000 Genomes Phase I integrated variant call set release version 3 as a reference panel.**

**Results:**

**After genotyping and imputation quality control procedures, data were available for 15 non‐HLA single‐nucleotide polymorphisms in 1,024 cases and 6,348 controls. We confirmed the known markers *ANKRD55* (meta‐analysis odds ratio [OR] 0.80; *P* = 2.8 × 10^−13^)** **and *BLK* (OR 1.13; *P* = 7.0 × 10^−6^) and identified new and specific markers of anti‐CCP–negative RA (prolactin [*PRL*] [OR 1.13; *P* = 2.1 × 10^−6^] and *NFIA* [OR 0.85; *P* = 2.5 × 10^−6^]). Neither of these loci is associated with other common, complex autoimmune diseases.**

**Conclusion:**

**Anti‐CCP–negative RA and anti‐CCP–positive RA are genetically different disease subsets that only partially share susceptibility factors. Genetic polymorphisms located near the *PRL* and *NFIA* genes represent examples of genetic susceptibility factors specific for anti‐CCP–negative RA.**

Rheumatoid arthritis (RA) can be categorized as seronegative or seropositive, based on the presence or absence of anti–citrullinated protein autoantibodies (ACPAs). Most ACPA‐positive RA patients are positive for anti–cyclic citrullinated peptide (anti‐CCP) antibodies, a hallmark that is used to classify RA patients according to the 2010 American College of Rheumatology (ACR)/European League Against Rheumatism classification criteria 1. Although the two serotypes are not clinically distinguishable at diagnosis, the presence of anti‐CCP antibodies at baseline predicts the future development of erosive disease 2, 3. Debate continues as to whether anti‐CCP–positive and anti‐CCP–negative RA actually represent two distinct diseases, with a common clinical end point of synovial inflammation 4, 5, 6, 7, 8, 9.

The contribution of genetic factors to the susceptibility of each serotype was estimated to be equivalent in a small twin study 10; however, in a recent study using large population‐representative samples, the heritability calculation was revised and reported to be 50% for anti‐CCP–positive RA and 20% for anti‐CCP–negative RA 11. Although it was initially thought that HLA did not play a role in the etiology of anti‐CCP–negative RA 12, several studies have now shown its association with seronegative disease 5, 8, 13, 14. More recently, this association has been pinpointed to 2 amino acid positions within HLA molecules: position 11 of HLA–DRB1 and position 9 of HLA–B 15. Based on the small number of susceptibility loci identified within the HLA region and their relatively small effect sizes, it is unlikely that they completely explain the disease heritability of seronegative RA. Non‐HLA markers of anti‐CCP–negative RA are therefore likely to exist.

However, candidate gene and genome‐wide association studies (GWAS) of seronegative RA have identified few non‐HLA determinants of anti‐CCP–negative RA at confirmed levels of statistical significance. Most genetic associations specific for anti‐CCP–negative RA have been reported in single studies and have not been independently replicated. We have previously tested markers of anti‐CCP–positive RA for their association with anti‐CCP–negative RA 8 and reported that several anti‐CCP–positive RA susceptibility loci (e.g., *AFF3, CCR6, CCL21, IL2RA,* and *CD28*) were not shared with anti‐CCP–negative RA, while markers at *TNFAIP3, C5orf30, STAT4, ANKRD55, BLK,* and *PTPN22* were associated with both anti‐CCP–positive and anti‐CCP–negative RA. In addition, *CLYBL* 14, *SMIM21* 14, *SPP1* 16, *CLEC16A* 17, *IRF5* 18, and *DCIR* 19, 20 have been reported to be associated with anti‐CCP–negative RA. Of the markers reported to be associated with anti‐CCP–negative RA, only *CLYBL* 14, *SMIM21* 14, and *ANKRD55* 21 have been independently replicated or confirmed at genome‐wide levels of significance.

In a previous study of 11,475 RA cases and 15,870 controls genotyped for 129,464 markers using the ImmunoChip array, we identified 14 new RA susceptibility loci reaching a genome‐wide level of significance 21. In a subset analysis of the 3,339 anti‐CCP–negative RA cases, only rs71624119 mapping to intron 6 of *ANKRD55* reached genome‐wide significance levels outside the HLA region, although other variants showed suggestive levels of association. Therefore, in the present study, we tested these variants in an independent cohort of anti‐CCP–negative RA cases and controls to identify replicated susceptibility loci.

## PATIENTS AND METHODS

### Cohorts and patients

For the replication study, samples were obtained from 1,044 UK RA patients who did not take part in the ImmunoChip study, satisfied the 1987 ACR classification criteria for RA 22, and tested negative for anti‐CCP, as determined with the second‐generation CCP (CCP2) assay. These patients were selected from the Norfolk Arthritis Register, Rheumatoid Arthritis Medication Study, National Repository, and Biologics in RA Genetics and Genomics Study Syndicate (Table 1). Individuals from the Wellcome Trust Case Control Consortium 2 (WTCCC2) and from the arcOGEN study were used as controls. (See Appendix A for a list of arcOGEN Consortium members and their affiliations.) Individuals from the WTCCC2 who were used as controls in the ImmunoChip study were identified using identity by descent calculation and removed. The arcOGEN cohort comprised 7,410 unrelated patients with severe osteoarthritis (OA) 23. We excluded arcOGEN cases from Nottingham because those patients had only provided informed consent for participation in studies related to OA. Consequently, 5,459 arcOGEN cases were available as additional controls in our study. Informed consent was obtained from all patients, and ethics approval was obtained from all relevant institutional ethics committees.

**Table 1:** Summary of cohort characteristics^a^

Cohort	No. of patients	No. (%) female^b^	Criteria for diagnosis	Anti‐CCP–negative, %
BRAGGSS	296	225 (78)	1987 ACR criteria for RA	100
NR	24	23 (96)	1987 ACR criteria for RA	100
RAMS	119	80 (67)	1987 ACR criteria for RA	100
NOAR	585	369 (67)	1987 ACR criteria for RA	100
Total	1,024	–	–	–

a Anti‐CCP = anti–cyclic citrullinated peptide; BRAGGSS = Biologics in Rheumatoid Arthritis Genetics and Genomics Study Syndicate; ACR = American College of Rheumatology; RA = rheumatoid arthritis; NR =National Repository; RAMS = Rheumatoid Arthritis Medication Study; NOAR = Norfolk Arthritis Register.

b Percentage of the individuals for whom information on sex was available.

### Selection of single‐nucleotide polymorphisms (SNPs)

The patients and methods used to identify non‐HLA genetic polymorphisms associated with anti‐CCP–negative RA in the discovery study (ImmunoChip) have been described previously 21. Briefly, 3,339 anti‐CCP–negative cases (Supplementary Table 1 in ref. 21) were compared to 15,870 controls. Cases and controls were obtained from 6 different cohorts (UK, Swedish Epidemiological Investigation of Rheumatoid Arthritis, US, Dutch, Spanish, and Swedish Umea) and genotyped on the ImmunoChip platform (186 regions densely mapped with 44,973 individual SNPs between the densely mapped regions [singletons]). When the ImmunoChip array was designed, the densely mapped regions were included if they had previously shown strong evidence of association with at least one autoimmune disease, while less stringent evidence was available for singletons. Effect sizes were meta‐analyzed across the 6 cohorts as previously described 21. There were 1,000 anti‐CCP–negative RA cases and 8,430 controls originating from the UK on the ImmunoChip (UK ImmunoChip cohort). In order to select SNPs for the replication study presented here, we first excluded the HLA region (segment 25–35 Mb on chromosome 6); then, the SNP with the lowest association *P* value for anti‐CCP–negative RA was selected for every densely mapped region or for every linkage disequilibrium block (r^2^ = 0.8) between the regions. Finally, 2 sets of SNPs were selected, based on the following *P* thresholds: 1) SNPs with *P* < 1.0 × 10^−4^ for anti‐CCP–negative patients from the meta‐analysis of the 6 ImmunoChip cohorts; and 2) SNPs with *P* ≤ 3.0 × 10^−4^ for anti‐CCP–negative patients from the UK ImmunoChip cohort.

### Genotyping

Control data were available from both the WTCCC2, genotyped on Affymetrix version 6.0 and an Illumina 1.2M platform, and from arcOGEN samples, genotyped using Illumina Human610‐Quad BeadChips 23. Genotyping of anti‐CCP–negative RA cases in the replication cohort was performed using a Sequenom MassArray platform according to the manufacturer's instructions. SNPs that failed genotyping on Sequenom or accurate calling were first removed. Patients with a genotyping rate of <90% and SNPs with a success rate of <90% were removed, together with SNPs with a minor allele frequency (MAF) of <5%.

### Imputation

In order to impute genotypes for the WTCCC2, data sets from Affymetrix version 6.0 and the Illumina 1.2M platform were merged. Data from the WTCCC2 and arcOGEN were phased with SHAPEIT version 2 and imputed with IMPUTE2 using the 1000 Genomes Phase I integrated variant call set release version 3. Imputed probabilities were replaced by the best guess genotypes using the default threshold at 0.9. No INFO score cutoff was applied. Additional postimputation quality control was performed selectively for the replication SNPs using the same thresholds as applied for the genotyping quality controls described above for cases.

### Statistical analysis

#### Association study

Association testing with anti‐CCP–negative RA was performed with Plink version 1.07 24 using a basic allelic chi‐square test. The following strategy was applied to meta‐analyze the results from the discovery and replication studies: for the set of SNPs with *P* < 1.0 × 10^−4^ for anti‐CCP–negative patients from the meta‐analysis of the 6 ImmunoChip cohorts, results from the replication study were considered to be a seventh cohort and a fixed‐effects meta‐analysis with inverse variance weighting was applied across the 7 studies (or cohorts); for the set of SNPs with *P* ≤ 3.0 × 10^−4^ for anti‐CCP–negative patients from the UK ImmunoChip cohort, results from the replication study were combined for meta‐analysis only with results from the UK cohort of the ImmunoChip study.

#### Correction for multiple testing

The *P* values reported for the replication study were not corrected for multiple testing. Significance was assessed using a stringent Bonferroni correction for multiple testing. The number of independent tests was determined independently for each of the SNP sets described above after exclusion of positive controls (i.e., SNPs previously reported to be associated with anti‐CCP–negative RA: *BLK, STAT4, C5orf30,* and *ANKRD55*). We tested 5 SNPs from the UK ImmunoChip cohort (Table 2). None of the SNPs in this set had been previously reported to be associated with anti‐CCP–negative RA. Therefore, the Bonferroni corrected threshold for significance for this SNP set was 0.05/5 tests (*P* < 0.01). There were 8 SNPs from the meta‐analysis of the 6 ImmunoChip cohorts (Table 2); 3 SNPs in this SNP set (*C5orf30*, *BLK*, and *STAT4*) had previously been reported to be associated with anti‐CCP–negative RA. Therefore, the Bonferroni corrected threshold for significance for this SNP set was 0.05/(8 − 3) tests (*P* < 0.01). The Bonferroni corrected threshold for significance in this replication study was therefore set at *P* < 0.01 for any SNP tested. Considering the 2 sets of SNPs as independent experiments is a valid working hypothesis, since they barely overlap and they do not contain the same proportion of already identified markers of anti‐CCP–negative RA, which could be explained by population‐specific associations.

**Table 2:** Results of the replication study^a^

				Replication		Meta‐analysis (anti‐CCP–negative RA)^b^		ImmunoChip study (anti‐CCP–positive RA)^c^		Ratio of OR for anti‐CCP–positive RA to OR for anti‐CCP–negative RA from the ImmunoChip study^d^	
Chr.	SNP ID	Gene	Selected	OR (95% CI)	*P*	OR	*P*	OR	*P*	Ratio	*P*
5	rs71624119	*ANKRD55*	BOTH	0.85 (0.76–0.96)	6.0 × 10^−3^ e	0.80	2.8 × 10^−13^	0.84	1.7 × 10^−11^	1.07	8.2 × 10^−2^
6	rs10440835	*PRL*	IC	1.14 (1.04–1.26)	8.1 × 10^−3^ e	1.13	2.1 × 10^−6^	1.05	2.5 × 10^−2^	0.93	2.8 × 10^−2^
5	rs528092	*C5orf30*	IC	0.92 (0.83–1.01)	8.9 × 10^−2^	0.89	3.3 × 10^−6^	0.92	5.0 × 10^−4^	1.06	6.4 × 10^−2^
8	rs4840565	*BLK*	IC	1.12 (1.01–1.24)	2.9 × 10^−2^	1.13	7.0 × 10^−6^	1.09	3.4 × 10^−4^	0.97	0.33
16	rs28698667	*IL27*	IC	1.06 (0.97–1.17)	0.21	1.11	6.3 × 10^−5^	1.02	0.32	0.91	5.0 × 10^−3^
21	rs9979383	*RUNX1*	IC	0.96 (0.88–1.06)	0.47	0.91	2.0 × 10^−4^	0.91	3.2 × 10^−5^	1.02	0.55
18	rs16955629	*RAB31*	IC	1.03 (0.92–1.15)	0.62	1.11	2.1 × 10^−4^	1.02	0.34	0.91	1.0 × 10^−2^
2	rs10181656	*STAT4*	IC	1.04 (0.93–1.16)	0.50	1.11	2.2 × 10^−4^	1.14	2.4 × 10^−7^	1.00	0.90
2	rs888427	*CYBRD1*	IC	0.95 (0.86–1.05)	0.34	1.08	2.0 × 10^−3^	–	–	–	–
1	rs10489912	*NFIA*	UK	0.87 (0.79–0.96)	3.8 × 10^−3^ e	0.85	2.5 × 10^−6^	0.97	0.17	1.06	4.8 × 10^−2^
1	rs2249707	*SLAMF9*	UK	0.99 (0.90–1.10)	0.89	0.90	6.5 × 10^−3^	1.02	0.30	1.09	5.6 × 10^−3^
17	rs2689	*HNF1B*	UK	1.04 (0.94–1.14)	0.47	0.93	3.3 × 10^−2^	–	–	–	–
4	rs6533712	*CAMK2D*	UK	0.93 (0.85–1.02)	0.12	1.05	0.12	–	–	–	–
11	rs613587	*FLI1*	UK	0.89 (0.80–0.99)	3.2 × 10^−2^	1.05	0.15	–	–	–	–

a After quality control procedures, the genotypes of 14 single‐nucleotide polymorphisms (SNPs) were analyzed in the replication phase in 1,024 anti–citrullinated protein autoantibody (anti‐CCP)–negative rheumatoid arthritis (RA) cases and 6,348 controls. Chr. = chromosome; OR = odds ratio; 95% CI = 95% confidence interval.

b Meta‐analysis of discovery (ImmunoChip [IC]) and replication cohorts.

c Meta‐analysis across the 6 cohorts of the ImmunoChip study of the association with anti‐CCP–positive RA.

d A ratio of 1.0 indicates that the effect size is the same for anti‐CCP–positive RA and anti‐CCP–negative RA. The *P* value for the ratio indicates whether a genetic marker is significantly differentially associated between the 2 disease subsets. The comparison with anti‐CCP–positive RA was performed only if the direction of the effect size for anti‐CCP–negative RA was consistent between the discovery and replication studies.

e Significant after Bonferroni correction (*P* < 0.01).

#### Establishing the specificity of anti‐CCP–negative associations

When the direction of the effect size for anti‐CCP–negative RA was consistent between the discovery and replication studies, the effect size for anti‐CCP–negative RA was formally compared with the effect size for anti‐CCP–positive RA. A multinomial logistic regression was applied in the discovery ImmunoChip study to compute odds ratios (ORs), 95% confidence intervals (95% CIs), and *P* values for association between the minor allele of every SNP and either anti‐CCP–positive RA or anti‐CCP–negative RA, assuming additivity on the log‐odds scale. To test for differences between OR for anti‐CCP–positive RA and OR for anti‐CCP–negative RA, the linear combination β+ − β−, where β+ is log(OR for anti‐CCP–positive RA) and β− is log(OR for anti‐CCP–negative RA), was calculated, along with its standard error. The *P* value for the difference in association between anti‐CCP–positive RA and anti‐CCP–negative RA was then calculated. Statistical analysis was performed with Stata version 12.1 (StataCorp) at the High Performance Computing facility of The University of Manchester.

#### Calculation of a genetic risk score (GRS) and receiver operating characteristic (ROC) analysis

GRS calculation and ROC curve analysis (including the calculation of the area under the ROC curve [AUC]) were performed according to the method of Karlson and as described previously 25, 26. Briefly, the GRS was calculated as the sum of the risk allele counts, weighted by the natural logarithm of the OR. Since ORs are usually inflated in the discovery cohort (“winner's curse” effect), we computed the GRS using ORs calculated from the replication study presented here. The association of the GRS with anti‐CCP–negative RA was tested by logistic regression. Clinical usefulness was evaluated with ROC curve analysis and calculation of the AUC using Stata.

## RESULTS

### Selection of SNPs for the replication study

SNPs selected from the meta‐analysis of the 6 ImmunoChip cohorts and SNPs selected from the UK ImmunoChip cohort are presented in Table 3. SNP rs71624119 mapping to the *ANKRD55* locus was the only SNP that reached genome‐wide significance for anti‐CCP–negative RA in the ImmunoChip study (i.e., *P* below the threshold for genome‐wide significance at *P* = 5.0 × 10^−8^) and was the only SNP in common between the top hits from the meta‐analysis and from the UK ImmunoChip cohort (Table 3). This SNP was therefore used as a positive control in our replication study. Among the 13 SNPs selected from the UK ImmunoChip cohort, only *CLEC16A* had previously been reported to be associated with anti‐CCP–negative RA 17, but other SNPs/genes (for example *STAT4, BLK,* and *C5orf30*) were not among the best hits in this cohort. They were, however, among the 12 SNPs selected from the meta‐analysis across the 6 ImmunoChip cohorts. Other markers reported to be associated with anti‐CCP–negative RA (i.e., *PTPN22*) were not among the best hits from the meta‐analysis. The majority of the markers selected for replication (Table 3) are represented by SNPs lying outside regions densely genotyped on the ImmunoChip, i.e., outside regions expected to be associated with autoimmune diseases.

**Table 3:** Loci selected for replication from the ImmunoChip study^a^

Chr.	SNP ID	Gene	Fine mapped^b^	Selected^c^	Dropped^d^	Reason dropped	Minor allele	Major allele	Frequency in controls^e^	Frequency in anti‐CCP–negative RA^e^	OR	*P*
5	rs71624119	*ANKRD55*	Densely genotyped	IC/UK	No	–	A	G	0.25	0.20	IC: 0.79 UK: 0.77	IC: 6.23 × 10^−12^ UK: 1.20 × 10^−5^
5	rs528092	*C5orf30*	Densely genotyped	IC	No	–	C	T	0.33	0.30	0.87	1.09 × 10^−5^
2	rs888427	*CYBRD1*	Outside	IC	No	–	T	C	0.35	0.39	1.13	3.63 × 10^−5^
9	rs7857530	*TXNDC4*	Outside	IC	Yes	Incompatibility for multiplex	G	A	0.44	0.41	0.89	5.43 × 10^−5^
18	rs16955629	*RAB31*	Outside	IC	No	–	A	G	0.24	0.27	1.14	6.00 × 10^−5^
3	rs1875463	*SLC9A9*	Outside	IC	Yes	Primer design failed	T	C	0.24	0.21	0.87	7.09 × 10^−5^
16	rs28698667	*IL27*	Densely genotyped	IC	No	–	T	C	0.42	0.46	1.13	7.57 × 10^−5^
6	rs10440835	*PRL*	Outside	IC	No	–	T	C	0.33	0.36	1.12	8.00 × 10^−5^
7	rs16879645	*ELMO1*	Densely genotyped	IC	Yes	Incompatibility for multiplex	C	T	0.10	0.12	1.19	8.13 × 10^−5^
8	rs4840565	*BLK*	Densely genotyped	IC	No	–	C	G	0.27	0.31	1.13	8.53 × 10^−5^
6	rs9483788	*HBS1L*	Outside	IC	Yes	Failed to genotype	C	T	0.26	0.29	1.13	8.73 × 10^−5^
2	rs10181656	*STAT4*	Densely genotyped	IC	No	–	G	C	0.22	0.24	1.14	9.84 × 10^−5^
21	rs9979383	*RUNX1*	Outside	IC	No	–	C	T	0.37	0.33	0.89	9.90 × 10^−5^
16	rs79349411	*IRF8*	Densely genotyped	UK	Yes	Primer design failed	A	C	0.09	0.12	1.35	3.68 × 10^−5^
11	rs613587	*FLI1*	Outside	UK	No	–	G	A	0.28	0.33	1.23	5.26 × 10^−5^
22	rs117794103	*MTMR3*	Densely genotyped	UK	Yes	Failed at post‐genotyping QC	G	A	0.01	0.03	1.90	5.86 × 10^−5^
1	rs67087057	*DPH5*	Densely genotyped	UK	Yes	Failed at post‐imputation QC	T	A	0.33	0.37	1.22	6.13 × 10^−5^
6	rs1002475	*REV3L*	Densely genotyped	UK	Yes	Incompatibility for multiplex	T	C	0.44	0.49	1.20	1.20 × 10^−4^
1	rs10489912	*NFIA*	Outside	UK	No	–	A	C	0.42	0.38	0.83	1.65 × 10^−4^
1	rs2249707	*SLAMF9*	Outside	UK	No	–	C	T	0.32	0.28	0.82	1.68 × 10^−4^
17	rs2689	*HNF1B*	Outside	UK	No	–	A	T	0.48	0.44	0.84	1.75 × 10^−4^
2	rs116752433	*KIAA1841*	Densely genotyped	UK	Yes	Primer design failed	A	G	0.03	0.01	0.45	1.91 × 10^−4^
4	rs6533712	*CAMK2D*	Outside	UK	No	–	T	A	0.46	0.51	1.19	2.01 × 10^−4^
16	rs116899727	*CLEC16A*	Densely genotyped	UK	Yes	Failed to genotype	T	G	0.02	0.01	0.42	2.18 × 10^−4^
15	rs1444291	*AGBL1*	Outside	UK	Yes	Incompatibility for multiplex	C	T	0.27	0.23	0.81	2.41 × 10^−4^
8	rs4736558	*KCNQ3*	Outside	UK	Yes	MAF difference in controls	T	A	0.24	0.27	1.22	3.00 × 10^−4^

a Chr. = chromosome; OR = odds ratio; QC = quality control.

b Indicates whether the single‐nucleotide polymorphism (SNP) maps to one of the 186 densely mapped regions on the ImmunoChip (IC).

c IC indicates that the SNP was selected based on the *P* value of the meta‐analysis across the 6 cohorts of the IC, while UK refers to the list of SNPs from the UK ImmunoChip cohort.

d “Dropped” indicates SNPs not available for analysis.

e Minor allele frequency (MAF) in controls or patients with anti–cyclic citrullinated peptide (anti‐CCP)–negative rheumatoid arthritis (RA) in the UK samples from the ImmunoChip study.

### Genotyping results for anti‐CCP–negative RA cases and controls

DNA samples from UK anti‐CCP–negative RA patients (n = 1,044) were genotyped for 19 SNPs; 2 SNPs failed to genotype, and 1 was excluded in postgenotyping quality control. Twenty patient samples were excluded based on their low genotyping rate. After quality control, the genotypes for 16 SNPs were available in 1,024 anti‐CCP–negative RA patients (Table 1). The total genotyping rate was 99.95%, and all SNPs were in Hardy‐Weinberg equilibrium. After imputation and quality control procedures, 5,283 individuals who were diagnosed as having OA from the arcOGEN Consortium and 4,766 control individuals from the WTCCC2 with available genome‐wide genotypes were available as controls for the present study, with a total genotyping rate of 99.42%. Data for 15 of the 16 SNPs successfully genotyped in cases could be analyzed in controls (Tables 2 and 3).

Significant differences in MAFs between WTCCC2 and arcOGEN data sets for any of the 15 SNPs of interest could indicate either population stratification at these loci, a specific association with OA, or imputation error. It has previously been reported that there is very little population stratification within the UK 27, and when we compared the MAFs of the 15 SNPs of interest between the WTCCC2 and arcOGEN samples, only 1 SNP, rs4736558 on chromosome 8, showed any difference in MAF (uncorrected *P* = 0.010) (Table 4), and that SNP was removed. Therefore, we concluded that the WTCCC2 and arcOGEN samples could be merged and used together as controls. After removal of 3,701 WTCCC2 samples that were already used as controls in the ImmunoChip study, a total of 6,348 controls were available for analysis, together with 1,024 cases. Therefore, 8 SNPs from the ImmunoChip meta‐analysis and 5 SNPs from the UK ImmunoChip cohort were available for replication analysis.

**Table 4:** Comparison of MAFs of SNPs in the arcOGEN and WTCCC2 cohorts^a^

Chr.	SNP	MAF in arcOGEN cases (n = 5,283)	MAF in WTCCC2 controls (n = 4,766)	OR (95% CI)
1	rs10489912	0.42	0.42	1.01 (0.96–1.07)
1	rs2249707	0.31	0.31	1.00 (0.94–1.06)
2	rs888427	0.36	0.36	1.02 (0.96–1.08)
2	rs10181656	0.23	0.22	1.04 (0.98–1.11)
4	rs6533712	0.48	0.47	1.04 (0.99–1.10)
5	rs71624119	0.24	0.24	1.02 (0.95–1.09)
5	rs528092	0.33	0.33	1.00 (0.95–1.06)
6	rs10440835	0.34	0.33	1.03 (0.97–1.09)
8	rs4840565	0.28	0.27	1.02 (0.96–1.08)
8^b^	rs4736558	0.24	0.23	1.09 (1.02–1.16)
11	rs613587	0.30	0.29	1.04 (0.97–1.10)
16	rs28698667	0.41	0.42	0.98 (0.93–1.04)
17	rs2689	0.49	0.48	1.02 (0.96–1.08)
18	rs16955629	0.24	0.24	1.02 (0.96–1.09)
21	rs9979383	0.38	0.37	1.01 (0.95–1.07)

a WTCCC2 = Wellcome Trust Case Control Consortium 2; Chr. = chromosome; OR = odds ratio; 95% CI = 95% confidence interval.

b The single‐nucleotide polymorphism (SNP) rs4736558 on chromosome 8 was the only SNP that showed any difference in minor allele frequency (MAF) (uncorrected *P* value = 0.010) and was excluded from the analysis.

### Results of statistical analysis

Results of the analysis are presented in Table 2. As expected, rs71624119 (*ANKRD55*) was associated with anti‐CCP–negative RA (OR 0.85; *P* = 6.0 × 10^−3^) in the replication cohort (Table 2). Of the 14 SNPs tested, 10 showed a consistent direction of association between the discovery and replication data sets (OR consistently greater than or less than 1.0). Among these, 3 SNPs near *PRL, NFIA*, and *BLK* were associated with *P* < 0.05 in the replication data set. The significance of associations for SNPs near *PRL* and *NFIA* persisted after correction for multiple testing. The association of rs10440835 (near *PRL*) is very unlikely to be driven by the known association with HLA 15, since rs10440835 is not in linkage disequilibrium with the HLA region (r^2^ < 0.001 between rs10440835 and the HLA–DRB1*0401 tag SNP rs6910071, calculated in the ImmunoChip data set). All other associations shown in Table 2 are also independent of the HLA, since they arose from genetic markers that are not located on chromosome 6. The association statistics presented in Table 2 are unlikely to arise from low imputation quality, since most SNPs were directly genotyped in controls or had an INFO score of >0.97 if imputed (Table 5).

**Table 5:** Quality of imputation or genotyping of SNPs presented in Table 2^a^

			arcOGEN		WTCCC2		Anti‐CCP–negative RA cases
Chr.	SNP ID	Gene	INFO^b^	Type	INFO^b^	Type	Genotyping rate, %^c^
1	rs10489912	*NFIA*	1.00	Directly typed	1.00	Directly typed	99.8
1	rs2249707	*SLAMF9*	1.00	Directly typed	1.00	Directly typed	99.9
2	rs10181656	*STAT4*	0.99	Imputed	1.00	Imputed	100
2	rs888427	*CYBRD1*	1.00	Directly typed	1.00	Directly typed	99.9
4	rs6533712	*CAMK2D*	0.99	Imputed	1.00	Directly typed	100
5	rs71624119	*ANKRD55*	0.98	Imputed	0.97	Imputed	100
5	rs528092	*C5orf30*	1.00	Imputed	1.00	Imputed	100
6	rs10440835	*PRL*	0.99	Imputed	1.00	Directly typed	99.9
8	rs4840565	*BLK*	0.99	Imputed	1.00	Imputed	100
11	rs613587	*FLI1*	1.00	Directly typed	1.00	Directly typed	100
16	rs28698667	*IL27*	1.00	Directly typed	1.00	Imputed	99.8
17	rs2689	*HNF1B*	0.99	Imputed	1.00	Imputed	100
18	rs16955629	*RAB31*	1.00	Directly typed	1.00	Directly typed	100
21	rs9979383	*RUNX1*	1.00	Directly typed	1.00	Directly typed	99.9

a SNPs = single‐nucleotide polymorphisms; Chr. = chromosome; WTCCC2 = Wellcome Trust Case Control Consortium 2.

b Postimputation INFO score.

c Number of anti–citrullinated protein (anti‐CCP)–negative rheumatoid arthritis (RA) patients in the replication data set (UK) with available genotype information after quality control, divided by the total number of individuals (n = 1,024).

### Meta‐analysis

For SNPs selected from the UK ImmunoChip cohort, the meta‐analysis was performed between that cohort and the replication cohort, while for SNPs selected from the meta‐analysis of the 6 ImmunoChip populations, the meta‐analysis was performed between those 6 populations and the UK replication cohort, which was treated as a seventh population. Meta‐analysis increased the confidence in the true nature of the association of *ANKRD55* with anti‐CCP–negative RA (OR 0.80; 2.8 × 10^−13^) (Table 2). Similarly, the association statistics were strengthened for several SNPs, in particular for *PRL* (OR 1.13; *P* = 2.1 × 10^−6^) and *NFIA* (OR 0.85; 2.5 × 10^−6^), increasing the confidence that they represent true associations.

### Comparison of associations with anti‐CCP–negative RA and associations with anti‐CCP–positive RA

The comparison of the association of *ANKRD55* with anti‐CCP–positive samples and the association of *ANKRD55* with anti‐CCP–negative samples showed that the *ANKRD55* polymorphism is shared between the 2 serotypes (Table 2). The same was observed for *BLK*: the ratio of the effect size between the 2 serotypes was not significantly different from 1.00. However, *PRL* and *NFIA* SNPs were not associated with anti‐CCP–positive RA and were significantly differentially associated between the 2 serotypes, suggesting that they represent specific anti‐CCP–negative associations (Table 2).

### GRS and ROC analysis

**Figure 1:**
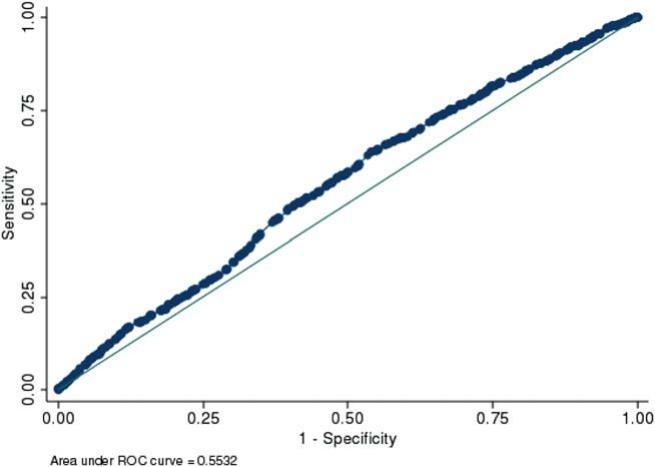
Receiver operating characteristic (ROC) curve analysis for a genetic risk score calculated for *ANKRD55*, *PRL*, *C5orf30*, *BLK*, *STAT4*, and *NFIA*. The area under the curve is 0.5532; clinical utility can therefore be achieved only if other markers (genetic and/or environmental) can be incorporated. Color figure can be viewed in the online issue, which is available at http://onlinelibrary.wiley.com/journal/doi/10.1002/art.39619/abstract.

A GRS was computed with all known and newly identified non‐HLA genetic markers of anti‐CCP–negative RA available in this study (Table 2): *ANKRD55*, *PRL*, *C5orf30*, *BLK*, *STAT4*, and *NFIA*. As expected, the GRS was associated with anti‐CCP–negative RA (OR 2.74 [95% CI 1.92–3.91]) (*P* = 2.41 × 10^−8^). The ROC curve is presented in Figure 1; the AUC was 0.55.

## DISCUSSION

The ImmunoChip study 21 represents the largest study of genetic markers of seronegative RA to date, but only the HLA region and the *ANKRD55* locus showed confirmed association in anti‐CCP–negative RA patients (i.e., below the threshold for genome‐wide significance at *P* = 5.0 × 10^−8^). However, there were a number of variants that showed suggestive levels of association, and here we report the results of a replication study in an independent group of RA cases and controls from the UK. We have identified 2 loci that show replicated evidence of association in anti‐CCP–negative disease.

The first locus, rs10440835, is located on chromosome 6 in the intergenic domain between the prolactin (*PRL*) and the neurensin 1 (*NRSN1*) gene. SNP rs10440835 is not known to be associated with anti‐CCP–positive RA at a genome‐wide level of significance, is situated 7 Mb away from the center of the HLA region, and is not in linkage disequilibrium with HLA. Therefore, the association of rs10440835 is unlikely to be confounded by HLA associations. Although the *PRL* region was not densely genotyped on the ImmunoChip, prolactin has been the focus of studies in RA over many decades 28. Prolactin plays a predominant role in lactation in the postpartum period, and parity and breastfeeding are both associated with RA susceptibility 29. Hyperprolactinemia is observed in a proportion of patients with RA 30, 31. Prolactin is secreted from the pituitary gland as well as other organs and cells, including lymphocytes. It acts both as a hormone and a cytokine to regulate the function of a variety of tissues, including immune cells and cartilage 32. The modification of prolactin levels has been suggested to have therapeutic potential in RA 32. Among the immunomodulatory functions of prolactin, this hormone has been reported to increase tumor necrosis factor expression in the peripheral CD14+ monocytes of patients with RA 33. However, no genetic association with RA susceptibility was found in the *PRL* region in the latest and largest RA susceptibility study published to date, comprising predominantly anti‐CCP–positive RA 34.

The second locus, rs10489912, is located on chromosome 1 in an intron of nuclear factor I/A (*NFIA*), a member of the NF‐1 family of transcription factors. *NFIA* has been shown to regulate the production, differentiation, and/or function of several immune cell subsets, including granulocytes 35, monocyte/macrophages 36, and CD314−CD158a+ natural killer cells 37, which are all immune cell subsets of the innate immune system. An association of the *NFIA* locus with a form of RA without antibodies is interesting, since the production of anti‐CCP antibodies requires the engagement of the adaptive immune system (T and B lymphocytes).

Interestingly, the majority of SNPs that had suggestive levels of association and were selected for replication testing from the ImmunoChip study mapped outside classical autoimmune‐related regions. These are unexpected findings because genetic markers of autoimmune diseases are largely shared with at least one other trait 38. Although our novel findings for anti‐CCP–negative disease (*PRL* and *NFIA*) are exceptions to the general trend of cross‐disease traits, our study provides evidence that they are unlikely to be false positives. *BLK*, *C5orf30*, and *STAT4* have already been reported to be associated with seronegative RA in independent studies, 10 of 14 SNPs showed a consistent direction of association, and the 2 newly identified markers, located near the *PRL* and *NFIA* genes, showed statistically significant evidence of independent replication.

The novel associations at *PRL* and *NFIA* add to the list of suggestive or confirmed anti‐CCP–negative RA susceptibility loci: *PTPN22, TNFAIP3, C5orf30, STAT4, BLK* 8, *SPP1* 16, *CLEC16A* 17, *IRF5* 18, *DCIR* 19, 20, *CLYBL* 14, *SMIM21* 14, and *ANKRD55* 21. However, only a few of those associations have been independently replicated or confirmed at genome‐wide levels of significance. With over 4,300 anti‐CCP–negative samples (in the discovery and replication cohorts together), the present study is the largest worldwide. If the genetic architecture of anti‐CCP–negative RA were similar to that of anti‐CCP–positive RA with regard to the total number of SNPs conferring susceptibility and their effect sizes, our study should have been as successful as equivalently well‐powered studies of anti‐CCP–positive RA, as performed ∼5 years ago.

However, it appears clear that the effect sizes detected in this study are small and the strength of association (*P* value) does not reach the threshold for genome‐wide significance. This could be partially explained by the fact that the genetic contribution to anti‐CCP–negative RA susceptibility is lower than the heritability of anti‐CCP–positive RA, but it is likely to also reflect the heterogeneity of anti‐CCP–negative RA 39. If anti‐CCP–negative RA comprises several different clinical subsets, each caused by different sets of SNPs, the identification of susceptibility SNPs without knowledge of the yet unidentified subsets 39 will be difficult, and limited to SNPs shared between the most prevalent subsets. The development of new genetic methodologies is therefore required to define genetically distinct disease subsets that cannot simply be classified clinically or based on serologic tests.

Ultimately, the identification of genetic markers of disease susceptibility will lead to personalized or stratified medicine in rheumatology. We have recently shown that the strongest genetic markers of RA susceptibility are also the strongest markers of RA course, severity, and mortality and possibly response to treatment with biologic agents 40. Therefore, the current identification of genetic susceptibility markers of RA subsets is likely to affect our future ability to guide clinical decisions based on the patient's personal genetic profile.

The goal of our study was to identify new genetic markers of anti‐CCP–negative RA and not to determine the cumulative predictive capacity of all already‐known markers, including those recently identified within the HLA region 15. Therefore, the genotypes at those loci were not all available in our replication cohort. Nonetheless, we were able to consider the effect and predictive capacity of a set of SNPs (*ANKRD55*, *PRL*, *C5orf30*, *BLK*, *STAT4*, and *NFIA*); though their aggregate association with anti‐CCP–negative RA is important (OR 2.74 [95% CI 1.92–3.91]) (*P* = 2.41 × 10^−8^), their predictive capacity remains very low (AUC 0.55). We have already shown for anti‐CCP–positive RA, for which the total number and effect size of genetic susceptibility markers identified so far are much larger than for anti‐CCP–negative RA, that 1) the predictive value of an aggregate GRS including all known markers is too low to be used in a clinical setting 26; and 2) the inclusion of non‐HLA SNPs into a GRS exclusively based on HLA markers did not significantly affect its predictive capacity 26. We show in the present study that the total number and effect sizes of anti‐CCP–negative loci are smaller than for anti‐CCP–positive RA; therefore, a much larger number of loci will need to be identified before their use can enter the clinic.

Our study therefore represents an important step in the development of genetically based algorithms for stratified medicine in rheumatology, since it highlights important limitations of the “single SNP” approach. We show that, for anti‐CCP–negative RA, as is the case for any other heterogeneous or rare autoimmune disease phenotype, our current experimental approaches reach their limitations. Even well‐powered studies using data from large international consortia fail to identify a sufficiently large number of susceptibility polymorphisms at genome‐wide significance that could explain a sufficiently large proportion of disease heritability to permit stratifications of individuals into different risk strata for personalized medicine. Therefore, the experimental approach of researchers in genetics of complex diseases needs to be changed from the identification of single SNPs (either through candidate gene approaches or GWAS) to statistical strategies identifying much larger sets of SNPs (possibly thousands) at the same time. Using a Bayesian inference analysis of the polygenic architecture of RA, Stahl et al 41 have already shown that, together, thousands of SNPs from RA GWAS would explain an additional 20% of disease risk, excluding known associated loci. Therefore, with new statistical techniques to come and further increase in sample sizes through international collaborations, the identification of much larger sets of anti‐CCP–negative RA associations and their use in computing clinically meaningful patient stratification algorithms for personalized medicine remains an achievable goal for the future.

Therefore, we can conclude that 1) non‐HLA genetic markers of anti‐CCP–negative RA do exist, 2) in general, their effect size is smaller than susceptibility markers for anti‐CCP–positive RA (meaning that larger sample sizes are required for detection), 3) they might not be preferentially located in autoimmune‐related genetic regions previously associated with other complex autoimmune diseases, 4) anti‐CCP–negative RA is likely to comprise several genetically distinct disease entities, and 5) increasing sample size and developing new analytical approaches are further required in the future before genetic and environmental diagnostic scores or scores predicting disease course, severity, or treatment response can enter clinical practice (stratified or personalized medicine).

Although further studies are required to definitively confirm the association reported here, our results provide evidence that anti‐CCP–negative and anti‐CCP–positive RA represent two genetically distinct disease subsets. The two disease subsets should therefore be investigated separately in future genetic studies aiming to identify pathogenic or causative pathways, which are likely to be different between the two serotypes.

## AUTHOR CONTRIBUTIONS

All authors were involved in drafting the article or revising it critically for important intellectual content, and all authors approved the final version to be published. Dr. Worthington had full access to all of the data in the study and takes responsibility for the integrity of the data and the accuracy of the data analysis.

### Study conception and design

Viatte, Massey, Bowes, Duffus, arcOGEN Consortium, Eyre, Barton, Worthington.

### Acquisition of data

Massey, Duffus, arcOGEN Consortium.

### Analysis and interpretation of data

Viatte, Bowes, Eyre, Barton, Worthington.

## APPENDIX A THE arcOGEN CONSORTIUM

Contributors from the arcOGEN Consortium are as follows: John Loughlin (Musculoskeletal Research Group, Institute of Cellular Medicine, Newcastle University, Newcastle‐upon‐Tyne, UK), Nigel Arden (Botnar Research Centre, University of Oxford, and Nuffield Orthopaedic Centre, Oxford, UK), Fraser Birrell (Musculoskeletal Research Group, Institute of Cellular Medicine, Newcastle University, Newcastle upon‐Tyne, and Northumbria Healthcare NHS Foundation Trust, Wansbeck General Hospital, UK), Andrew Carr (Botnar Research Centre, University of Oxford, and Nuffield Orthopaedic Centre, Oxford, UK), Panos Deloukas (William Harvey Research Institute, Barts and The London School of Medicine and Dentistry, Queen Mary University, London, UK and Princess Al‐Jawhara Al‐Brahim Centre of Excellence in Research of Hereditary Disorders [PACER‐HD], King Abdulaziz University, Jeddah, Saudi Arabia), Michael Doherty (Academic Rheumatology, School of Medicine, University of Nottingham, Nottingham, UK), Andrew W. McCaskie (Division of Trauma and Orthopaedic Surgery, Department of Surgery, University of Cambridge, Cambridge, UK and Musculoskeletal Research Group, Institute of Cellular Medicine, Newcastle University, Newcastle‐upon‐Tyne, UK), William E. R. Ollier (Centre for Integrated Genomic Medical Research, University of Manchester, Manchester, UK), Ashok Rai (Worcestershire Acute Hospitals NHS Trust, Worcester, UK), Stuart H. Ralston (Centre for Genomic and Experimental Medicine, Institute of Genetics and Molecular Medicine, University of Edinburgh, Edinburgh, UK), Tim D. Spector (Department of Twin Research and Genetic Epidemiology, King's College London, London, UK), Ana M. Valdes (Academic Rheumatology, School of Medicine, University of Nottingham, Nottingham, UK), Gillian A. Wallis (Wellcome Trust Centre for Cell Matrix Research, University of Manchester, Manchester UK), J. Mark Wilkinson (Department of Oncology and Metabolism, University of Sheffield, Sheffield, UK), and Eleftheria Zeggini (Wellcome Trust Sanger Institute, Wellcome Genome Campus, Hinxton, UK).
